# Systemic lupus erythematosus presenting with eosinophilic enteritis: a case report

**DOI:** 10.1186/1752-1947-5-235

**Published:** 2011-06-25

**Authors:** Mehrnaz Asadi Gharabaghi, Pejman Abdollahi, Mohammad Kalany, Masoud Sotoudeh

**Affiliations:** 1 Department of General Internal Medicine, Imam Khomeini Hospital Complex, Tehran University of Medical Sciences, Tehran, Iran; 2 Imam Khomeini Hospital Complex, School of Medicine, Tehran University of Medical Sciences, Tehran, Iran; 3 Department of Gastroenterology, Imam Khomeini Hospital Complex, School of Medicine, Tehran University of Medical Sciences, Tehran, Iran; 4 Department of Pathology, School of Medicine, Tehran University of Medical Sciences, Tehran, Iran

## Abstract

**Introduction:**

Systemic lupus erythematosus (SLE) is a multisystem disorder that may present with various symptoms. It may involve the gastrointestinal tract in a variety of ways; some of the most well-known ones are transaminitis, lupus mesenteric vasculitis, lupus enteritis and mesenteric vascular leakage. We describe a case of a patient with SLE who presented with a five-month history of diarrhea caused by eosinophilic enteritis. To the best of our knowledge, there are few cases reported in the literature of patients with SLE who initially present with chronic diarrhea due to eosinophilic enteritis.

**Case presentation:**

A 38-year-old Persian Iranian woman was admitted with a five-month history of diarrhea and abdominal pain. A physical examination showed nothing abnormal. Initially, she had only lymphopenia and mild eosinophilia. No autoimmune or infectious etiology was detected to justify these abnormalities. A thorough evaluation was not helpful in finding the etiology, until she developed a scalp lesion similar to discoid lupus erythematosus. Computed tomography showed small bowel wall thickening. Briefly, she manifested full-blown SLE, and it was revealed that the diarrhea was caused by eosinophilic enteritis.

**Conclusion:**

Considering SLE in a patient who presents with chronic diarrhea and lymphopenia may be helpful in earlier diagnosis and therapy. This is an original case report of interest to physicians who practice internal medicine, family medicine and gastroenterology.

## Introduction

Systemic lupus erythematosus (SLE) is the prototypical autoimmune disease caused by auto-antibodies that recognize self-antigens as foreigners. These pathogenic antibodies are produced against the components of the cell nucleus. The abnormal immune response results in target tissue injury, and the ensuing inflammatory reaction deranges various organ functions. Nearly every organ system may be involved in the disease course. It may present initially with non-specific symptoms or atypical manifestations, so the diagnosis may present a considerable challenge at the early stages [1-3]. In this way, it is not surprising that a patient who is initially diagnosed with acute inflammatory demyelinating polyneuropathy will later be found to have SLE [4]. SLE may involve the gastrointestinal tract in a variety of ways. Some of the most common manifestations are dyspepsia, hepatitis, mesenteric vasculitis, enteritis and mesenteric vascular leakage [5-8]. However, to our knowledge, there are few cases of the disease in which the patient initially presented with chronic diarrhea due to eosinophilic enteritis. Herein we report the case of 38-year-old woman who presented with a five-month history of diarrhea and abdominal pain and was also diagnosed with SLE.

## Case presentation

**Figure 1:**
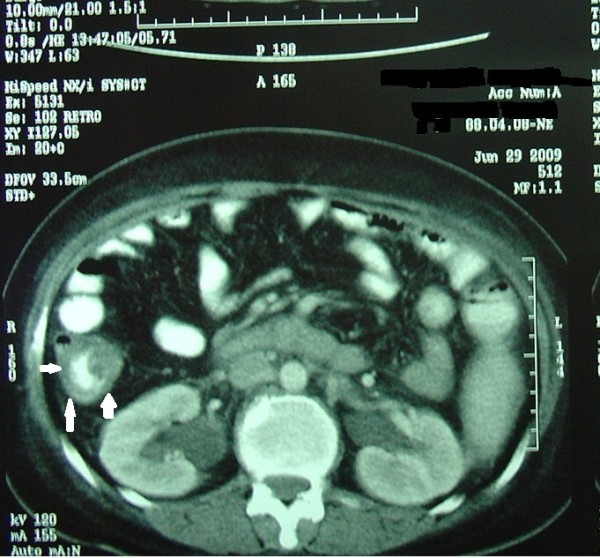
**Section of abdominal scan with oral contrast obtained at the time of admission showing increased thickness of the ascending colon wall**.

A 38-year-old Persian Iranian woman was admitted to our hospital to be evaluated for abdominal pain and diarrhea. Overall, she had been well until five months before her presentation. Thereafter she noticed a vague, constant pain over her abdomen. At the time of her admission to another hospital, the work-up culminated in a laparatomy. Her right ovary contained a hemorrhagic cyst that was resected. It was a benign ovarian cyst. She was then discharged. However, her abdominal pain persisted. A few days later she noticed frequent bowel movements with large amounts of watery stool each time. Occasionally, a sense of flushing also bothered her. The diarrhea persisted for more than two weeks without any change in spite of fasting or changes in food intake. She also experienced anorexia and weight loss. She underwent a through work-up at another hospital. Computed tomography (CT) of her abdomen was performed, which revealed thickening of the ascending bowel wall (Figure 1). Thereafter she was referred to our hospital to undergo further follow-up to rule out carcinoid syndrome.

She was a young woman concerned about her disease. Except for diarrhea and abdominal pain, she did not complain about fever, fecal incontinence or extra-intestinal manifestations. Her physical examination revealed pale conjunctivae and an enlarged spleen suggested by a dull percussion note on Traube's semilunar space. There were also several groups of slightly enlarged cervical and inguinal lymph nodes that were soft, mobile and non-tender.

She had no family history of inflammatory bowel disease or celiac disease. On the basis of the results of stool analyses and microbiologic studies, her condition was diagnosed as non-inflammatory diarrhea.

Since she had a CA-125 level to a high of 138 U/l (normal, up to 37 U/l), histological reexamination of tissues removed during her prior surgery was performed. The result was the same as the previous finding. The elevated level of CA-125 was attributed to a benign ovarian cystic disorder. Routine blood studies identified normochromic normocytic anemia, lymphopenia (hemoglobin level, 11.0 g/dl; lymphocyte count, 960/μl) and hypoalbuminemia (2.8 g/dl). There was no elevation in her erythrocyte sedimentation rate (25 mm/hour) or C-reactive protein level. Her blood chemistry showed no sign of electrolyte, hepatic or other metabolic disturbances. Neither nutritional deficiency nor blood loss was identified as the contributing factor to her anemia. Her serum ferritin level was 379 ng/ml (reference interval, 18 n g/ml to 341 ng/ml). An examination of a blood smear verified the presence of lymphocytopenia. Ultrasonography of the abdomen revealed an ovarian cyst in the left ovary measuring 3 cm × 5 cm in addition to a slightly enlarged spleen; otherwise, the findings were normal.

The most frequent cause of non-inflammatory chronic diarrhea among Iranians is celiac disease (CD) [9]. However, serologic biomarkers and endoscopic and histologic surveys of the duodenum were both inconsistent with CD. Biopsy specimens of the normal-appearing distal colon were taken. Her histological diagnosis inveighed against the presence of microscopic colitis. Her thyroid function tests were normal, too. The presence of diarrhea, abdominal pain and occasional flushing mandated screening for peptide hormones. Neuroendocrine tumors such as carcinoids might be considered. However, the results of the hormone assay disproved the existence of such tumors.

On the basis of our patient's medical symptoms, the differential diagnosis included SLE, intestinal lymphoma, acquired immunodeficiency syndrome (AIDS) and post-mucosal lymphatic obstruction. CT of her abdomen was performed with both oral and IV contrast agents to rule out structural or occult inflammatory intestinal disorders.

A bone marrow biopsy was evaluated but yielded no evidence of lymphoma. HIV antibody and SLE serologic biomarkers were checked. The results of the studies were pending at the time that she was discharged with instructions to return the following week.

After one week, she returned to our hospital complaining of new-onset painful lesions on her scalp. Her diarrhea and vague generalized abdominal pain persisted. She was admitted to our hospital for the second time.

There were several discrete erythematous plaques over her scalp containing circumscribed areas of alopecia. The lesions were similar to those seen in patients with discoid lupus erythematosus (DLE) or lichen planus. A skin biopsy confirmed the histologic diagnosis of DLE.

The results of HIV and fluorescence anti-nuclear antibody tests were negative, but anti-SSA/Ro antibody was detected in the blood in low titer. Her anti-double-stranded DNA (anti-dsDNA) antibody level was strongly positive at 5 IU/ml (> 1.1 was considered positive).

Peripheral eosinophilia was also present. Her eosinophil count was 780/μl, and her total white blood cell count was 4,610/μl. There was no evidence of parasitic infection on the basis of repeated examination of stool samples and serology tests to detect strongyloidosis, toxocariasis, trichinellosis, liver flukes and so on. She reported no history of allergic disorders or intake of over-the-counter drugs.

**Figure 2:**
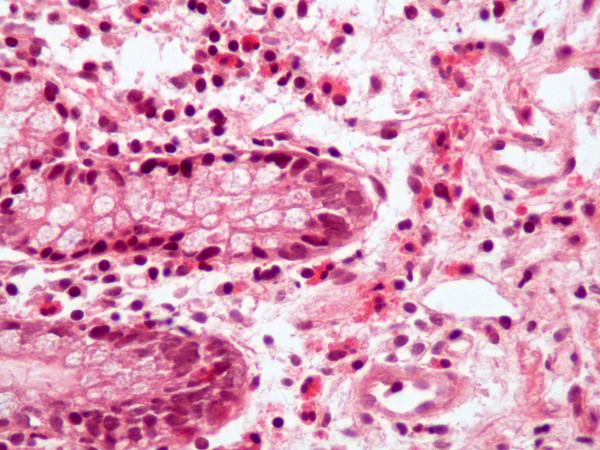
**Photomicrograph of colonic mucosa showing aggregation of eosinophils in deeper areas of the lamina propria between the tip of the mucosal glands and muscularis mucosa (hematoxylin and eosin stain; original magnification, × 320)**.

**Figure 3:**
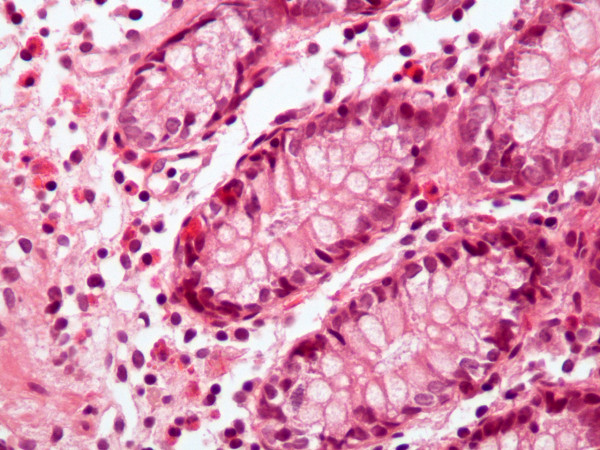
**Photomicrograph of colonic mucosa showing aggregation of eosinophils and exocytosis in the mucosal glandular epithelium (hematoxylin and eosin stain; original magnification, × 320)**.

CT of her abdomen demonstrated only bowel wall thickening; otherwise, the findings were normal. The results were consistent with an inflammatory bowel disorder such as Crohn's disease. The radiologist believed that intestinal lymphoma would be an alternative diagnosis. However, the clinical diagnosis rendered was lupus enteritis. Because of the discrepancies between the clinical and radiological diagnoses, she underwent total colonoscopy for the second time. Multiple biopsy specimens were also obtained from the ileum. The histologic findings were consistent with eosinophilic enteritis (Figures 2 and 3). The other possibilities, such as lymphoma, intestinal lymphangiectasia, vasculitis and inflammatory bowel disease, were excluded.

During her hospital stay, she developed proteinuria to a high of 4,800 mg/day. She was diagnosed with SLE because her symptoms met the criteria for lymphocytopenia, proteinuria, DLE and positive anti-dsDNA antibodies. A kidney biopsy was performed. The results of a histopathological study showed class V lupus nephritis. Considering the involvement of two significant organs (renal and gastrointestinal), she was treated by bolus therapy with three daily pulses of methylprednisolone at a dose of 1,000 mg/day followed by 0.5 mg/kg/day prednisolone. A few days later she no longer experienced diarrhea or abdominal pain. In this way, we reached the final diagnosis of eosinophilic enteritis in the context of SLE that caused the patient to develop anorexia, abdominal pain and diarrhea.

## Discussion

Diarrhea lasting more than four weeks mandates that the physician excludes the presence of serious underlying pathologies. The patient's medical history, physical examination and routine blood studies often help in establishing the mechanism of diarrhea. Hematologic clues such as lymphocytopenia narrow the extent of the differential diagnosis. Congenital intestinal lymphangiectasia or acquired lymphatic obstruction caused by a tumor may be responsible for the co-existence of chronic diarrhea and lymphocytopenia [10]. Other possible diagnoses include diarrhea caused by opportunistic infection in patients with AIDS [11].

Lymphoma may also involve the gastrointestinal tract in different ways. Both Hodgkin's and non-Hodgkin's lymphoma may invade the gastrointestinal tract. Two of the most common forms are mantle cell lymphoma and mucosa-associated lymphoid tissue lymphoma. Primary intestinal lymphomas may present with abdominal pain, symptoms due to intestinal obstruction, bleeding and diarrhea. Histologic examination of tissue obtained from the involved area often results in a proper diagnosis [12].

Eosinophilia and diarrhea may occur because of parasitic infections, neoplasia, collagen vascular disorders and eosinophilic gastroenteritis. Rheumatoid arthritis, allergic angiitis and peri-arteritis nodosa also may cause eosinophilia and gastrointestinal involvement [13].

Immune lymphocytopenia is also a characteristic feature of SLE that may be accompanied by diarrhea in a variety of ways. Lupus enteritis and eosinophilic enteritis may cause diarrhea in patients with SLE. Sometimes the diarrhea develops as a consequence of treatment complications, such as opportunistic infections occurring after cytotoxic therapy [5,7,8].

Eosinophilic enteritis is an infiltrative disorder that is characterized by patchy or diffuse eosinophilic infiltration of the intestinal wall. It rarely involves the entire intestinal tract. It usually presents as a distinct syndrome such as eosinophilic esophagitis, gastritis and enteritis. Dysphagia, dyspepsia and diarrhea have frequently been reported as the initial symptoms. Less frequently the disease may present with a focal mass, protein-losing enteropathy, ascites and even gastric outlet obstruction. The disease may develop in the context of another pre-existing disease such as CD or overlap syndrome. The disease responds well to corticosteroids; yet, there are cases that require no treatment because of the mild, non-interfering nature of the disease [14,15].

## Conclusion

To date, according to our knowledge, there have been few case reports of SLE presenting with diarrhea due to eosinophilic enteritis. Considering the disease in the differential diagnosis of patients presenting with diarrhea, eosinophilia and lymphopenia may be helpful for practicing physicians who are frequently confronted with chronic diarrhea cases. Therefore, this original case report may be of interest to internists, family physicians and gastroenterologists. The issue that should be taken into account is the following: SLE is similar to many autoimmune rheumatic diseases and may present atypically, lack a pathognomonic feature and run a dynamic course.

## Abbreviations

AIDS: acquired immunodeficiency syndrome; CD: celiac disease; CT: computed tomography; DLE: discoid lupus erythematosus; SLE: systemic lupus erythematosus.

## Consent

Written informed consent was obtained from the patient for publication of this case report and any accompanying images. A copy of the written consent is available for review by the Editor-in-Chief of this journal.

## Competing interests

The authors declare that they have no competing interests.

## Authors' contributions

MA was the main physician who cared for the patient and prepared the main draft of the manuscript. PA was the medical student who prepared the patient's medical history and physical examination. MS was the pathologist who reviewed the histological evaluation of the samples. KM was the gastroenterologist who performed the endoscopic procedures. All authors approved the final manuscript.
